# Anion-Directed Assembly of a Bimetallic Pd/Ag Nanocluster: Synthesis, Characterization, and HER Activity

**DOI:** 10.3390/molecules30020404

**Published:** 2025-01-18

**Authors:** Yu-Rong Ni, Rugma Thekke Pangal, Michael N. Pillay, Tzu-Hao Chiu, Samia Kahlal, Jean-Yves Saillard, C. W. Liu

**Affiliations:** 1 Department of Chemistry, National Dong Hwa University, Hualien 97401, Taiwan; 410512035@gms.ndhu.edu.tw (Y.-R.N.); rugmaramanunni95@gmail.com (R.T.P.); niven.sa@gmail.com (M.N.P.); a0930612399@gmail.com (T.-H.C.); 2 CNRS, ISCR, University of Rennes, UMR 6226, F-35000 Rennes, France; samia.kahlal@univ-rennes1.fr

**Keywords:** palladium, nanocluster, silver, hydride, hydrogen evolution

## Abstract

Palladium-doped silver nanoclusters (NCs) have been highlighted for their unique physicochemical properties and potential applications in catalysis, optics, and electronics. Anion-directed synthesis offers a powerful route to control the morphology and properties of these NCs. Herein, we report a novel Pd-doped Ag NC, [Pd(H)Ag_13_(S){S_2_P(O*^i^*Pr)_2_}_10_] (**PdHAg_13_S**), synthesized through the inclusion of sulfide and hydride anions. This NC features a unique linear S-Pd-H axis enclosed in a 4-5-4 stacked arrangement of silver atoms. The distinctive hydride environment was characterized by NMR spectroscopy, and the total structure was determined by single-crystal X-ray diffraction (SCXRD) and supported by computational studies. Mass spectrometry and X-ray photoelectron spectroscopy (XPS) further confirmed the assigned composition. This unique construct exhibits promising hydrogen evolution reaction (HER) activity. Our findings highlight the potential of anion-directed synthesis for creating novel bimetallic NCs with tailored structures and catalytic properties.

## 1. Introduction

The pursuit of atomically precise nanomaterials has led to the rapid expansion of nanocluster (NC) research [1,2,3]. These ultrasmall metal clusters, typically composed of a few to hundreds of atoms, bridge the gap between individual atoms and bulk materials. Unlike their bulk counterparts, NCs exhibit unique size-dependent properties arising from quantum confinement effects and the high proportion of surface atoms [4]. Among bimetallic systems, the combination of palladium and silver has long been exploited for its resistance to tarnish, a common problem with traditional sterling silver. On the nanoscale, the combination of metals can lead to synergistic effects, where the resulting properties are not merely a sum of the individual components but expanded functionalities compared to their monometallic counterparts. The controlled synthesis of Pd-doped Ag NCs with well-defined structures—for example, [PdAg_24_(SR)_18_]^2−^, [PdAg_28_(SR)_18_(PPh_3_)_4_]^2+^, and others—via metal exchange or co-reduction methods has been reported [5,6,7,8]. Achieving precise control over the size, composition, and atomic arrangement is crucial for tailoring their properties and optimizing their performance. Anion-directed synthesis has emerged as a powerful tool for manipulating the structure and composition of NCs. Anions are not limited to capping agents, but can actively direct the arrangement of metal atoms as templates, influencing the overall morphology of the NC [9,10,11,12]. Halides have provided this service, and a recent publication highlights their crucial role in forming vertex-sharing Pd and Pt-doped clusters [13,14,15]. The selective use of sulfides in assembling atomically precise nanoclusters was first exploited by Fenske and colleagues, who reported a series of large metallic frameworks and exploited the cooperative stabilization of chalcogens and ligands in large silver assemblies very early on [16,17,18]. Reports of discrete sulfide acting as bridging ligands in Pd/Ag complexes of the type [Ag_2_Pd_2_Cl_2_(dppf)_2_-(µ_3_-S)_2_] (dppf = 1,1′-bis(diphenylphosphin)ferrocene) also indicate the viability of the strategy [19,20]. Various NC morphologies have been identified due to the versatility of sulfide [16,17,18,19,20,21,22], including the recently reported [PdAg_16_(S)_2_{S_2_P(OR)_2_}_12_], wherein two sulfide anions were successfully incorporated [23]. However, the combination of two different anions is rare, and this report highlights the combination of sulfide and hydride. Considering the ability of hydrides to occupy interstitial sites within Pd/Ag metallic frameworks, we pursued the isolation of a NC containing both hydride and sulfide [24,25].

We report a novel molecule, [Pd(H)Ag_13_(S){S_2_P(O*^i^*Pr)_2_}_10_] (**PdHAg_13_S**), featuring a distinct linear S-Pd-H axis encapsulated by a silver cage and dithiophosphate (dtp) passivating layer. A suite of characterization techniques, including NMR spectroscopy, single-crystal X-ray diffraction (SCXRD), and computational studies, are applied to elucidate the NC’s composition, structure, and bonding. Furthermore, we explore the catalytic activity of this novel construct for the hydrogen evolution reaction (HER), a key process in sustainable energy production.

## 2. Results and Discussion

**Scheme 1:**
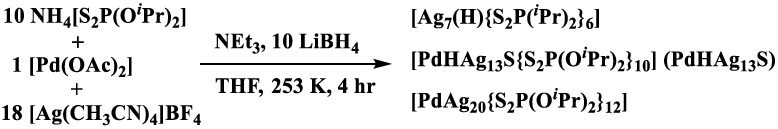
Synthetic methodology for incorporation of sulfide and hydride.

Earlier, we noted the isolation of the first sulfide-centered Pd/Ag of the type [Pd_6_Ag_14_(S){S_2_P(OR)_2_}_12_], indicating the cleavage of the P-S bond from the dithiophosphate and the in situ generation of the sulfide anion. We subsequently refined the method and determined that the addition of triethylamine can facilitate P-S bond cleavage, and selectively incorporated two sulfide ions in the formation of [PdAg_16_(S)_2_{S_2_P(OR)_2_}_12_] [23]. NC synthesis is extremely sensitive to stoichiometric ratios, reaction times, and conditions [23,24,25,26,27]. In this report, we further tuned the stoichiometric ratios of the precursors and reaction time (Scheme 1), yielding a novel NC with two anions of the type [Pd(H)Ag_13_(S){S_2_P(O*^i^*Pr)_2_}_10_] (**PdHAg_13_S**), with known by-products [Ag_7_(H){S_2_P(O*^i^*Pr)_2_}_12_] and [PdAg_20_{S_2_P(O*^i^*Pr)_2_}_12_] (Scheme 1) [27,28].

**Figure 1:**
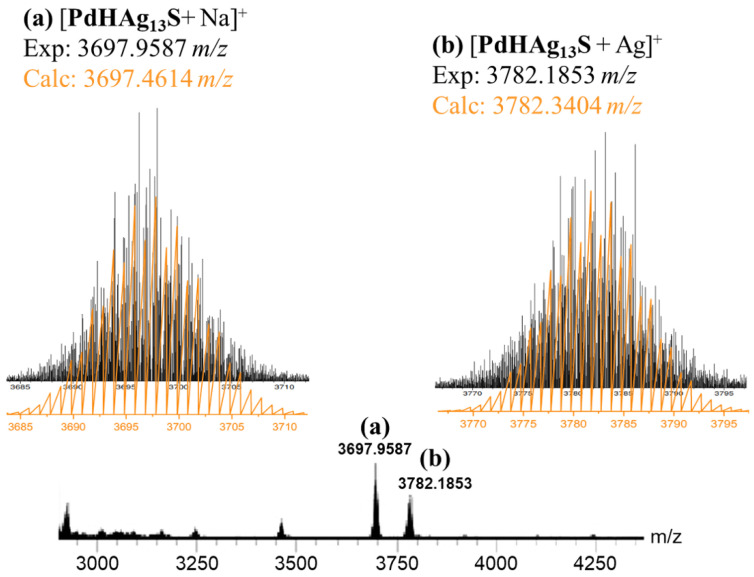
The ESI-TOF-MS spectrum of **PdHAg_13_S**. The positive ion peaks with sodium (**a**) and silver (**b**) adduct peaks in black, with simulated isotopic distribution overlaid in orange.

The purity and composition of the isolated NC was confirmed by mass spectroscopy. The ESI-TOF-MS spectrum indicates the formation of both silver and sodium adducts during ionization, with peaks obtained at *m*/*z* 3782.1853 Da assigned to [**PdHAg_13_S**+Ag]^+^ (Calc: *m*/*z* 3782.3404 Da) and *m*/*z* 3697.9587 Da for [**PdHAg_13_S**+Na]^+^ (Calc: *m*/*z* 3697.4614Da) (Figure 1). The cluster is prone to decomposition during the ionization process, with the peaks assigned to several smaller silver clusters (Figure S1). The XPS analysis further establishes the cluster’s composition and verifies that Pd(0) and the Ag approaching Ag(I) are based on their distinctive binding energies (Figure S2).

**Figure 2:**
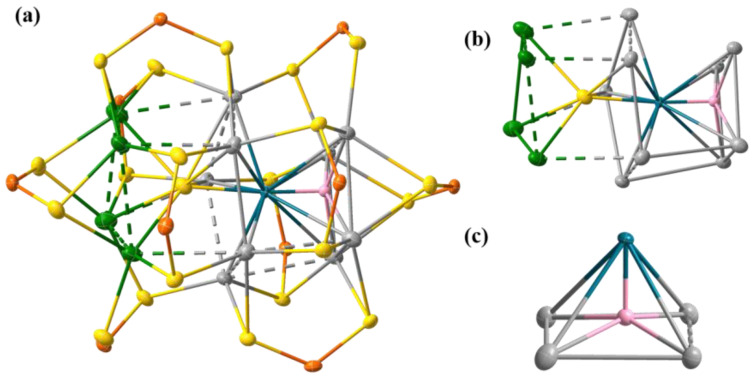
(**a**) Molecular representation of **PdHAg_13_S** (ligand carbon and hydrogen atoms omitted for clarity) ellipsoid drawn at 35%, metallic framework (**b**), and interstitial hydride (**c**). (Color code: Pd, blue; Ag_ker_, gray; Ag_out_, green; S, yellow; P, orange; H, pink).

In the solid state, **PdHAg_13_S** exhibits a unique structural motif centered around a pentagonal arrangement of five silver atoms (Figure 2a). The metallic framework can be viewed as a sandwiched structure with a central Ag_5_ motif flanked by Pd and S atoms and further capped by two Ag_4_ motifs (Figure 2b). Notably, the interstitial µ_5_ hydride resides within a square pyramidal cavity formed by one Pd and four Ag atoms (Figure 2c), an arrangement observed in previously reported PdH-doped silver superatoms. The axis connecting the S, Pd, and H atoms (S-Pd-H) is nearly perpendicular to the plane of the Ag_5_ motif, deviating by 5.92°. This moderate tilt contributes to the *C*_1_ symmetry observed in the solid state. The Pd-S bond, measuring 2.259(7) Å, is notably shorter than in traditional molecular complexes. The passivating layer contains three η_3_ (μ_2_, μ_1_) and eight η_4_ (μ_2_, μ_2_) ligands, resulting in an overall neutral configuration.

DFT-optimized geometry was performed on a simplified model for **PdHAg_13_S** [Pd(H)Ag_13_(S)(S_2_PH_2_)_10_] to gain insights into its structure and electronic properties. The optimized geometry obtained from these calculations is in good agreement with the structure determined by single-crystal X-ray diffraction (SCXRD) (see Table 1 and the corresponding XYZ file in Table S1). Analysis of the computed data supports the view of describing the whole NC as made of a quasi-linear [SPdH]^3−^ 14-electron complex (Pd-H = 1.664 Å) stabilized by a shell of 13 Ag(I) centers (Table 2 and Figure S5). Two significantly different Ag(I) environments can be distinguished as nine Ag(I) atoms coordinated to the Pd(0) atom (Ag_ker_) and four outer Ag(I) atoms (Ag_out_) anchored by a μ_7_ sulfide. NAO atomic charges (Table 2) support this assignment. Four Ag_ker_ atoms form a square pyramid, with the Pd atom encapsulating an interstitial μ_5_ hydride. The Pd-H and Pd-S bonds exhibit significant covalent character, as evidenced by their relatively large Wiberg bond indices (WBIs, Table 2). The Ag-S(dtp) bonds also show substantial covalent character. In addition to these covalent interactions, metallophilic interactions between the silver atoms contribute to the stability of the NC, as exemplified by the Ag…Ag WBI values, which are typical for this type of interaction (Table 2). The Kohn–Sham orbital diagram (Figure S5) provides further insights into the electronic structure. The highest occupied molecular orbitals (HOMOs) are primarily composed of 4d(Pd) and 3p(S), whereas the lowest unoccupied molecular orbitals (LUMOs) are 5p(Pd) and 5s/5p(Ag_ker_). Time-dependent DFT (TD-DFT) calculations were performed to simulate the UV–vis spectrum. The calculated UV–vis spectrum is in reasonable agreement with the experimental spectrum (Figure S6). It shows a low-energy band attributed to the HOMO-1 → LUMO+2, HOMO-1 → LUMO+1, and HOMO-2 → LUMO transitions, of 4d(Pd)/3p(S) → 5s/5p(Ag_ker_) in nature. These transitions involve charge transfer from the Pd and S atoms to the Ag atoms.

**Figure 3:**
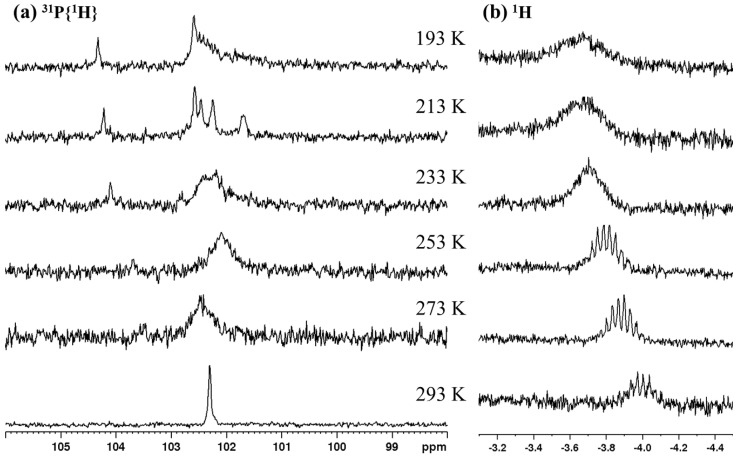
VT–NMR spectra of **PdHAg_13_S** in (CD_3_)_2_CO. (**a**) ^31^P{^1^H} NMR and (**b**) ^1^H NMR spectra.

The ^31^P{^1^H} NMR spectrum displays a single resonance at 102.32 ppm at ambient temperature (Figure S3a), indicating a time-averaged equivalence of all phosphorus atoms resulting from the dynamic processes. However, upon cooling to 213 K, the peak splits into five distinct resonances, correlating to non-equivalent phosphorus environments observed in the solid state (Figure 3a). The disorder observed in the solid state is also present in the solution, as evidenced by ^1^H NMR data. A multiplet resonance was observed at −3.98 ppm (Figure S3b), confirming the presence of a hydride. Notably, the integration ratio of the ^1^H NMR spectrum further supports the assignment of ten dtp ligands per single hydride and correlates to isopropyl substituents. The multiplicity observed indicates that the hydride couples to several Ag nuclei due to the dynamic nature of the hydride. This observation is consistent with the dynamic flipping of the S-Pd-H axis observed in the solid-state structure. Furthermore, the variable temperature NMR (VT-NMR) experiments indicate that the hydride resonance remains broad at low temperatures (213 K), suggesting residual mobility (Figure 3b). In solution, the dynamic behavior of this encapsulated unit, coupled with the fluxionality of the ligand shell, is expected to play a crucial role in the cluster’s effectiveness as an electrocatalyst for hydrogen evolution reactions (HER).

**Figure 4:**
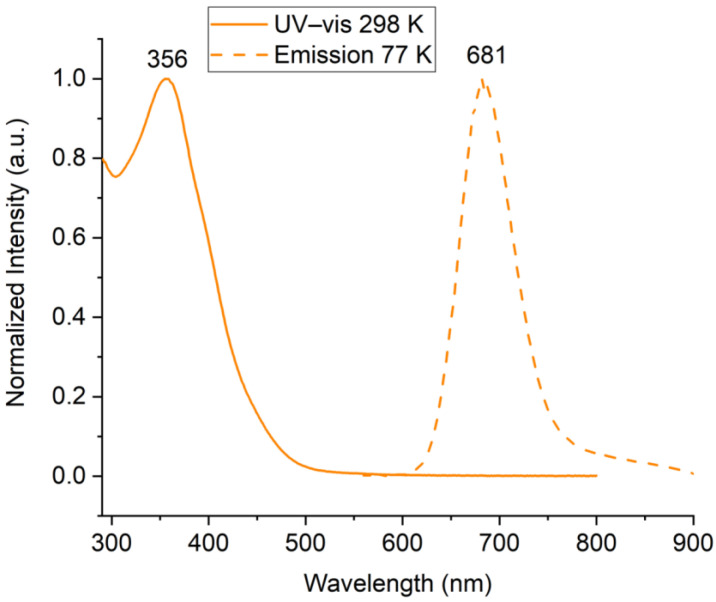
Optical spectra for **PdHAg_13_S** recorded in 2-MeTHF.

A key goal in tailoring a NC is the tuning of optical properties, and incorporating sulfide yields an emissive NC. Comparing previously reported silver-rich palladium NCs, **PdHAg_13_S** has the shortest emissive wavelength reported for chalcogen-passivated NCs [23,24,25]. The optical spectrum for **PdHAg_13_S**, with an absorption maximum centered at 356 nm (Figure 4), is similar to the previously reported [PdAg_16_S_2_{S_2_P(O*^i^*Pr)_2_}_12_], with 365 nm [23]. However, the emission wavelength is blue shifted (λem = 681 nm, τ = 121.1 µs) (Figure S4) compared to [PdAg_16_S_2_{S_2_P(O*^i^*Pr)_2_}_12_] (λem = 808 nm, τ = 92.3 µs).

**Figure 5:**
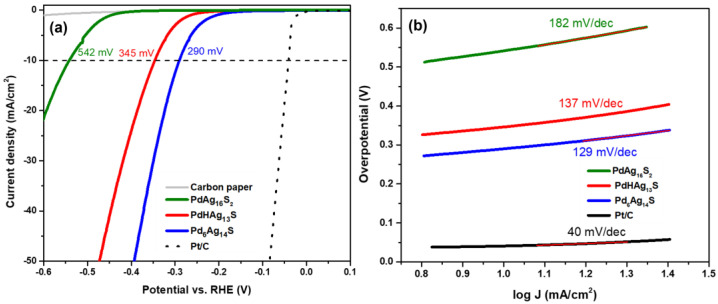
(**a**) LSVs recorded at 1 mV s^−1^ for **Pd_6_Ag_14_S**, **PdHAg_13_S**, **PdAg_16_S_2_**, Pt/C, and carbon paper; (**b**) Tafel slopes of **Pd_6_Ag_14_S**, **PdHAg_13_S**, **PdAg_16_S_2_**, and Pt/C.

**Figure 6:**
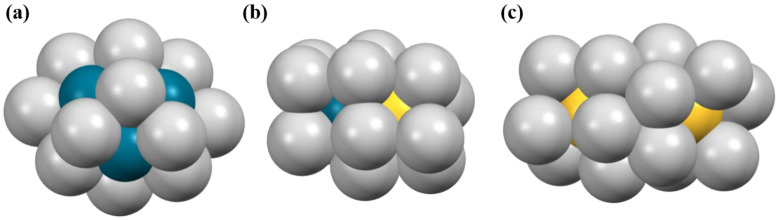
The space-filling models of (**a**) **Pd_6_Ag_14_S**, (**b**) **PdHAg_13_S**, and (**c**) **PdAg_16_S_2_** with the dtp ligand omitted. (Color code: Pd, blue; Ag, gray; S, yellow).

Hydrogen is widely regarded as a highly promising energy source because of its environmental sustainability and zero-emission properties. The electrocatalytic HER plays a crucial role in renewable and clean energy initiatives. Noble metals like Pt and Pd are known for their exceptional electrocatalytic efficiency in HER, largely due to their optimal hydrogen binding energy, favorable Gibbs free energy for atomic hydrogen adsorption, and low activation barriers for hydrogen desorption. Although silver is typically considered one of the least reactive metals in HER processes, the cost-effectiveness and abundance of silver-based nanomaterials have sparked interest in investigating their potential for hydrogen evolution [29,30,31]. The HER activity of **PdHAg_13_S** was compared with a related sulfide containing [Pd_6_Ag_14_(S){S_2_P(OPr)_2_}_12_] (**Pd_6_Ag_14_S**) [26], and [PdAg_16_(S)_2_{S_2_P(O*^i^*Pr)_2_}_12_] (**PdAg_16_S_2_**) [23], by linear sweep voltammetry (LSV) in a three-electrode electrochemical cell containing an argon saturated 0.5 M H_2_SO_4_ solution (Figure 5a). **Pd_6_Ag_14_S** and **PdHAg_13_S** exhibit higher HER activity than **PdAg_16_S_2_**. The overpotentials to reach a current density of −10 mAcm^−2^ were found to be −290, −345, and −542 mV for **Pd_6_Ag_14_S**, **PdHAg_13_S**, and **PdAg_16_S_2_**, respectively, indicating that **Pd_6_Ag_14_S** is the most efficient H_2_-producing electrocatalyst in the series. The Tafel slope was used to evaluate the kinetics of the HER, which were 129, 137, and 182 mV/dec, for **Pd_6_Ag_14_S**, **PdHAg_13_S**, and **PdAg_16_S_2,_** respectively (Figure 5b). A lower Tafel slope typically indicates more efficient electron transfer, leading to faster reaction rates. The **Pd_6_Ag_14_S** and **PdHAg_13_S** clusters, with Tafel slopes of 129 and 137 mV/dec, have relatively more efficient HER kinetics compared to **PdAg_16_S_2_** (182 mV/dec). Furthermore, the Tafel slopes of **Pd_6_Ag_14_S** and **PdHAg_13_S** are closer to the theoretical Tafel slope of the Volmer step (120 mV/dec), indicating that proton adsorption is the rate-determining step. The improved efficiency of the silver sites compared to monometallic clusters [32] is due to the synergistic effect of the Pd atom encapsulated by the silver metallic framework. This concept has already been explored in the literature to enhance the HER activity of nanoclusters. Studies such as that by Choi et al. [33] have shown that doping gold nanoclusters with metals like Pd and Pt significantly enhances HER activity by lowering hydrogen adsorption-free energy. Jin’s group [34] also demonstrated that doping Au_38_(SR)_24_ with metals boosted HER performance, and Shen et al. [35] found that Pt alloying improved the performance of Ag_29_ clusters. These findings highlight the role of doped metals in improving HER performance, even when buried within the core of the cluster. In our case, while Pd may not act as the primary catalytic site, it likely plays a key role in the overall HER activity of the alloyed nanocluster. This is supported by space-filling models (ligands omitted) of Pd_6_Ag_14_S, PdHAg_13_S, and PdAg_16_S_2_, as shown in Figure 6a–c. Pd_6_Ag_14_S exposes more Pd sites, which correlates with its higher HER activity. On the other hand, PdHAg_13_S exposes fewer Pd sites, and PdAg_16_S_2_ exposes none, which align with the observed trend in HER activity, decreasing in the same order. However, in comparison to open Pd sites, such as those found in [PdHCu_11_{S_2_P(O*^i^*Pr)_2_}_6_(C≡CPh)_4_] [36]_,_ the performance is significantly lower, further highlighting that defect engineering or ligand modifications may reveal the reactions on Pd of these clusters, which potentially enhance their activity for HER.

## 3. Materials and Methods

All reactions were conducted using standard Schlenk protocols under an inert N_2_ environment. All chemicals were used as supplied from commercial sources, including palladium(II) acetate (PdOAc_2_, 98%) and lithium borohydride (LiBH_4_, 2 M in THF). [Ag(MeCN)_4_]BF_4_ [37] and NH_4_[S_2_P(O*^i^*Pr)_2_] [38] were prepared according to previous reports. NMR and VT-NMR spectra were recorded on a Bruker Advance II 400 spectrometer, operating at 400 MHz for ^1^H and 161.97 MHz for ^31^P{^1^H}. ESI-TOF-MS spectra were recorded on a Fison Quattro Bio-Q (Fisons Instruments, VG Biotech, UK). The XPS spectra were recorded using a PHI 5000 VersaProbe-Scanning ESCA Microprobe on an X-ray Photoelectron Spectrometer. The absorption spectrum was recorded on an Agilent Cary-60 spectrometer. The PL spectra and lifetime decays were measured in an EPR tube with a liquid cryogenic system at 77 K and were recorded on a HORIBA FluoroMax plus spectrometer.

SCXRD analysis.

Paratone oil was used to coat and mount a single crystal onto a glass fiber. Data were recorded with a graphite mono-chromated Mo-Kα radiation source (λ = 0.71073 Å) fitted on a Bruker APEX II CCD diffractometer operating at 100 K. Data analysis was done with SADABS and SAINT, for adsorption corrections on raw data frames [39,40]. SHELXL-2018/3 package integrated into SHELXL/PC V6.14 [41,42,43] was used to solve the structure, which was then refined using least-squares versus F^2^. Anisotropic refinements were applied to all non-hydrogen atoms. Structure refinement details are summarized below in Table 3.

Computational Methods.

The geometry optimization was performed by density functional theory (DFT) calculations with the Gaussian16 package [44], with a BP86 functional [45,46] and the all-electron Def2-TZVP basis set from the EMSL Basis Set Exchange Library [47,48]. The optimized geometries were characterized as true minima through vibrational analysis. The natural atomic orbital (NAO) charges and the Wiberg bond indices (WBI) were computed with the NBO6.0 program [49,50] on single-point calculations performed with the BP86 functional and the Def2-SVP basis set for computational limitations [51,52]. The UV–visible transitions were calculated by means of time-dependent DFT (TD-DFT) calculations, with the CAM-B3LYP functional [53] and the Def2-TZVP basis set. The UV–visible spectra were simulated from the computed TD-DFT transitions and their oscillator strengths by using the Multiwfn program [54]. The compositions of the molecular orbitals were calculated using the AOMix program [55].

Electrocatalytic Measurements.

Electrode preparation: The catalyst ink was prepared by sonicating the mixture of the nanocluster and CH_2_Cl_2_ for 20 min. A total of 3 nmol of the catalyst ink was loaded onto a piece of carbon paper (1 cm^2)^, which was air-dried overnight before use.

Electrochemical measurements: Measurements were performed on an Admiral Instruments: Squidstat Plus Potentiostat. A three-electrode electrochemical cell consisting of a catalyst-immobilized carbon paper electrode, a Pt foil anode (3 cm^2^), and an Ag/AgCl reference electrode was used. The electrolyte solution of 0.5 M H_2_SO_4_ was degassed with Ar gas for 30 min before the electrochemical measurements. For comparison, a benchmark Pt/C electrode was prepared by drop casting 200 μL of a catalyst ink—made by dispersing 10 mg of Pt/C with 200 μL of 5 wt% Nafion solution, in 800 μL of isopropyl alcohol—onto carbon paper. Electrode potential measured was converted to the RHE scale using the following equation: E_RHE_ = E_Ag/AgCl_ + 0.197 + 0.059 pH (1).

Synthesis of [Pd(H)Ag_13_(S){S_2_P(O*^i^*Pr)_2_}_10_], **PdHAg_13_S**.

[NH_4_][S_2_P(O*^i^*Pr)_2_] (0.26 g, 1.13 mmol), [Pd(OAc)_2_] (0.025 g, 0.11 mmol), [Ag(CH_3_CN)_4_]BF_4_ (0.74 g, 2.06 mmol), NEt_3_ (100 µL, 0.717 mmol), and THF (20 mL) were combined in a reaction flask for 20 min. LiBH_4_ (0.3 mL, 0.59 mmol) was added with vigorous stirring at 253 K. The color of the solution gradually changed from orange to brown. After 4 h, the mixture was evaporated, and the residue was extracted with DCM and washed with water. Column chromatography with Al_2_O_3_ was used to purify the yellow target product with mixed solvent DCM and ether (*v*:*v* = 5:1), [Pd(H)Ag_13_(S){S_2_P(O*^i^*Pr)_2_}_10_], 0.86% yield. The by-products are eluted with solvent ratios (10:1) and (1:1) to yield [Ag_7_(H){S_2_P(O*^i^*Pr)_2_}_6_] in 18.6% and [PdAg_20_{S_2_P(O*^i^*Pr)_2_}_12_] in 31.6%, based on Pd. ^1^H NMR (400 MHz, (CD_3_)_2_CO, δ, ppm, 298 K): 4.94 (septet, CH, 20H), 1.39 (d, CH_3_, 120H); −3.98 (^1^*J*_HAg_ = 13.2 Hz). VT ^1^H NMR (400 MHz, (CD_3_)_2_CO, δ, ppm, 213 K): −3.66. ^31^P{^1^H} NMR (161.97 MHz, (CD_3_)_2_CO, δ, ppm, 298 K): 102.32. VT ^31^P{^1^H} NMR (161.97 MHz, (CD_3_)_2_CO, δ, ppm, 213 K): 104.21, 102.57, 102.46, 102.25, 101.69. ESI-MS (*m*/*z*): exp. 3782.1853 (calc.3782.3404 for [PdHAg_13_S +Ag]^+^) and exp. 3697.9587 (calc. 3697.4614 for [PdHAg_13_S + Na]^+^). UV–vis [λ_max_ in nm, (ε in M^−1^cm^−1^)]: 356 (66023). XPS (Calc.: Pd%0.95, Ag%12.38, S%20.00, P%9.52, C%57.14): Pd%0.94, Ag%12.40, S%20.07, P%9.50, C%57.09, and (binding energy, eV): Ag 3d_5/2_, 367.55; Ag 3d_3/2_, 373.55; Pd 3d_5/2_, 337.31; Pd 3d_3/2_, 342.57.

## 4. Conclusions

This study successfully demonstrated the anion-directed synthesis of a novel palladium-doped silver nanocluster featuring a unique linear S-Pd-H unit which can be described as a [SPdH]^3−^ anionic complex encapsulated within. Incorporating sulfide and hydride anions was crucial in directing the morphology. Comprehensive characterization, including NMR spectroscopy, ESI-MS, single-crystal X-ray diffraction (SCXRD), and computational studies, provided detailed insights into the NC’s composition, structure, and electronic properties. The NC exhibits promising catalytic activity for the hydrogen evolution reaction (HER), underscoring the potential of anion-directed synthesis as a powerful strategy for creating bimetallic NCs with tailored structures and properties for catalytic applications. Future research will explore the detailed mechanism of HER catalyzed by this NC and investigate the effects of ligand modification and alloying with other metals on its catalytic performance. Ultimately, this work opens new avenues for the design and synthesis of bimetallic nanoclusters with enhanced properties.

## Figures and Tables

**Table 1:** Comparison of experimental and computed distances (Å).

NC	SXRD	DFT
Pd-S	2.259(7)	2.315
Pd-Ag_ker_	2.778(9)–3.021(7) avg. 2.872(8)	2.848–3.086 avg. 2.956
Ag_ker_-Ag_ker_	2.893(6)–3.114(6) avg. 2.989(6)	2.988–3.155 avg. 3.051
Ag_out_-Ag_out_	3.043(7)–3.156(7) avg. 3.090(7)	3.120–3.190 avg. 3.155
Ag_ker_-S	2.665(12)–2.915(12) avg. 2.777(12)	2.779–2.845 avg. 2.812
Ag_out_-S	2.568(12)–2.903(9) avg. 2.745(9)	2.786–2.975 avg. 2.881

**Table 2:** Natural atomic orbital (NAO)-computed atomic charges and Wiberg bond indices (WBI).

Atom	NAO Charge	Bond	WBI
Pd	−0.58	Pd-H	0.373
H	−0.42	Pd-S	0.303
S	−1.28	Ag-H	0.057
Ag_ker_(avg)	0.68	Pd-Ag_ker_(avg)	0.069
Ag_out_(avg)	0.72	Ag_ker_-S(avg)	0.087
		Ag_out_-S(avg)	0.057
		Ag-Ag(avg)	0.039
		Ag-S(dtp)(avg)	0.141

**Table 3:** Crystal data and refinement of **PdHAg_13_S**.

Compound		[PdHAg_13_S{S_2_P(O*^i^*Pr)_2_}_10_]	
Chemical formula		C_60_H_141_Ag_13_O_20_P_10_PdS_21_	
CCDC		2411564	
Formula weight		3674.39	
Crystal system, space group		Monoclinic	C2/c
a, Å	α, deg	54.746(3)	90
b, Å	β, deg	17.9162(9)	99.799(2)
c, Å	γ, deg	27.3550(14)	90
Volume, Å^3^		26439	
Z		8	
ρcalcd, g·cm^−3^		1.846	
μ, mm^−1^		2.510	
Temperature, K		100(2)	
θmax, deg./Completeness, %		1.198/100	
Reflections collected/unique		140003/23299	
Restraints/parameters		364/1176	
R1 ^a^, wR2 ^b^ [I > 2σ(I)]		0.0381, 0.0751	
R1 ^a^, wR2 ^b^ (all data)		0.0510, 0.0804	
Goodness of fit		1.051	
Largest diff. peak and hole, e/Å^3^		2.418 and −1.748	

^a^ *R*1 = Σ||*F_o_*|-|*F_c_*||/Σ|*F_o_*|. ^b^ *wR*2 = {Σ[*w*(*F_o_*^2^ − F_c_^2^)^2^]/Σ[*w*(*F*_o_^2^)_2_]}^1/2^.

## Data Availability

Crystallographic data for PdHAg_13_S has been deposited at the Cambridge Crystallographic Data Centre under [CCDC 2,411,564] and can be obtained from www.ccdc.cam.ac.uk/data_request/cif (accessed on 22 December 2024). Computational details (including coordinate file), X-ray structure analyses, NMR spectra, electrochemical data, ESI-MS data, and luminescence decay curves are available in the Supplementary Information.

## Supplementary Materials

The following supporting information can be downloaded at: https://www.mdpi.com/article/10.3390/molecules30020404/s1.
