# Supplementary material for: Anion-Directed Assembly of a Bimetallic Pd/Ag Nanocluster: Synthesis, Characterization, and HER Activity
Source: Molecules. 2025 Jan 18;30(2):404. doi: 10.3390/molecules30020404 (PMC11767447; doi:10.3390/molecules30020404)
Supplement: Supplementary file 1 [file molecules-30-00404-s001.zip › molecules-3415902-supplementary.pdf]

Supporting Information

Anion-Directed Assembly of a Bimetallic Pd/Ag Nanocluster: Synthesis, Characterization, and HER activity

Yu-Rong Ni[1], Rugma T P[1] (a), Michael N. Pillay[1] (a), Tzu- Hao Chiu[1] (a), Samia Kahlal[2](b), Jean-Yves Saillard[2]* (b) C. W. Liu.[1]*

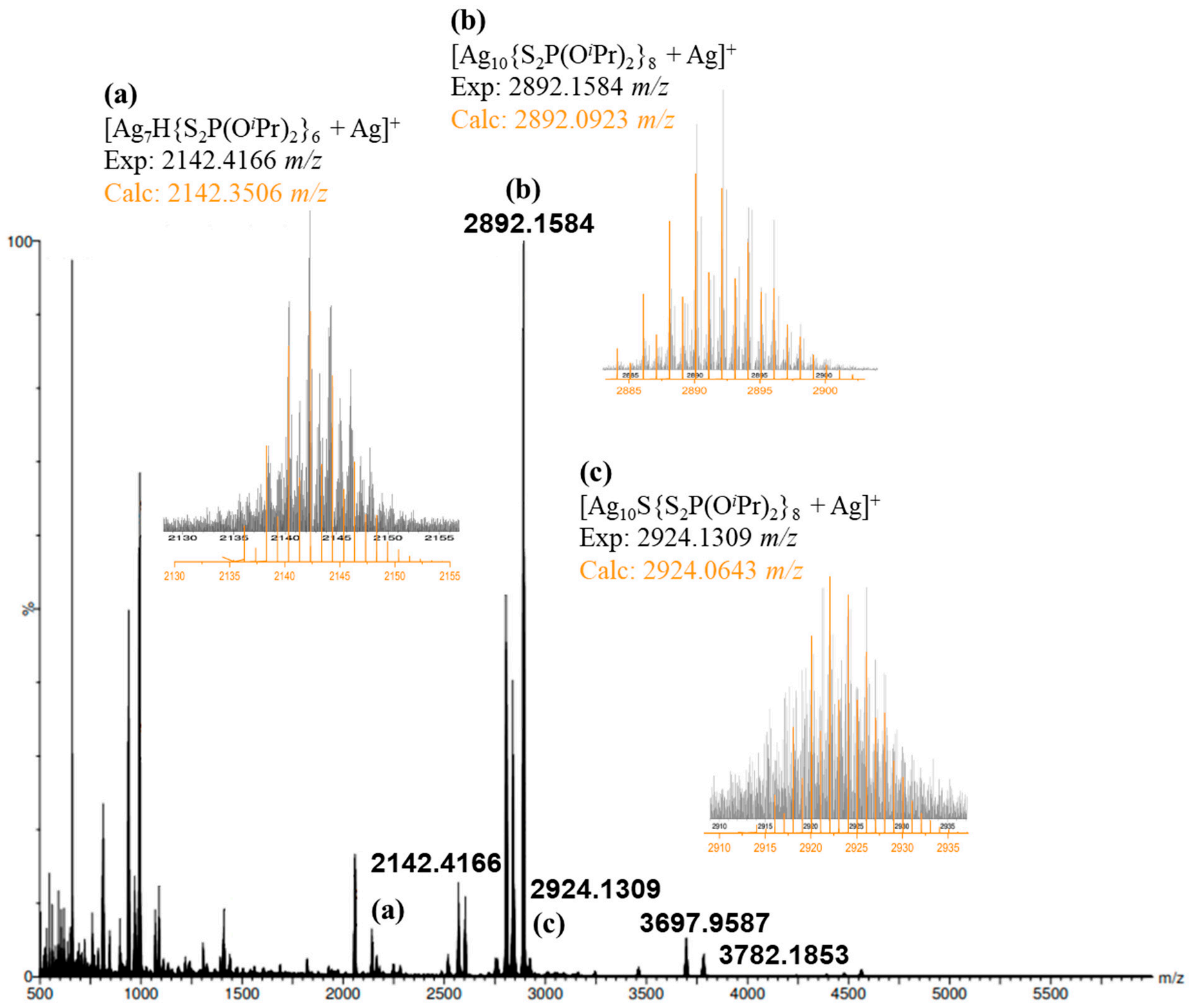

Figure S1. The full ESI-TOF-MS spectrum of **PdHAg$_{13}$S**. (a)-(c) The decomposition fragment peaks of **PdHAg$_{13}$S**.

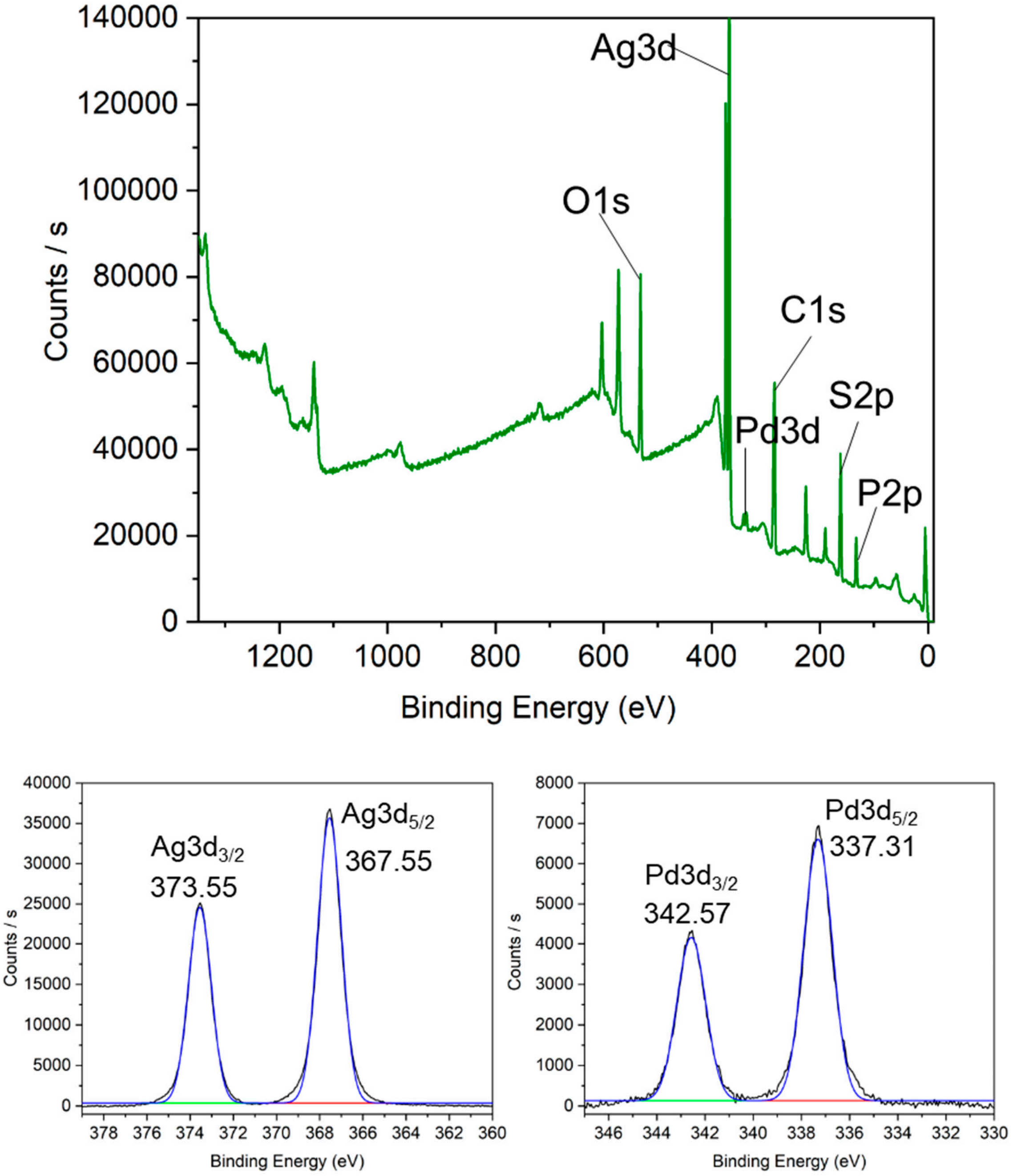

Figure S2. XPS analysis of **$PdHAg_{13}S$**.

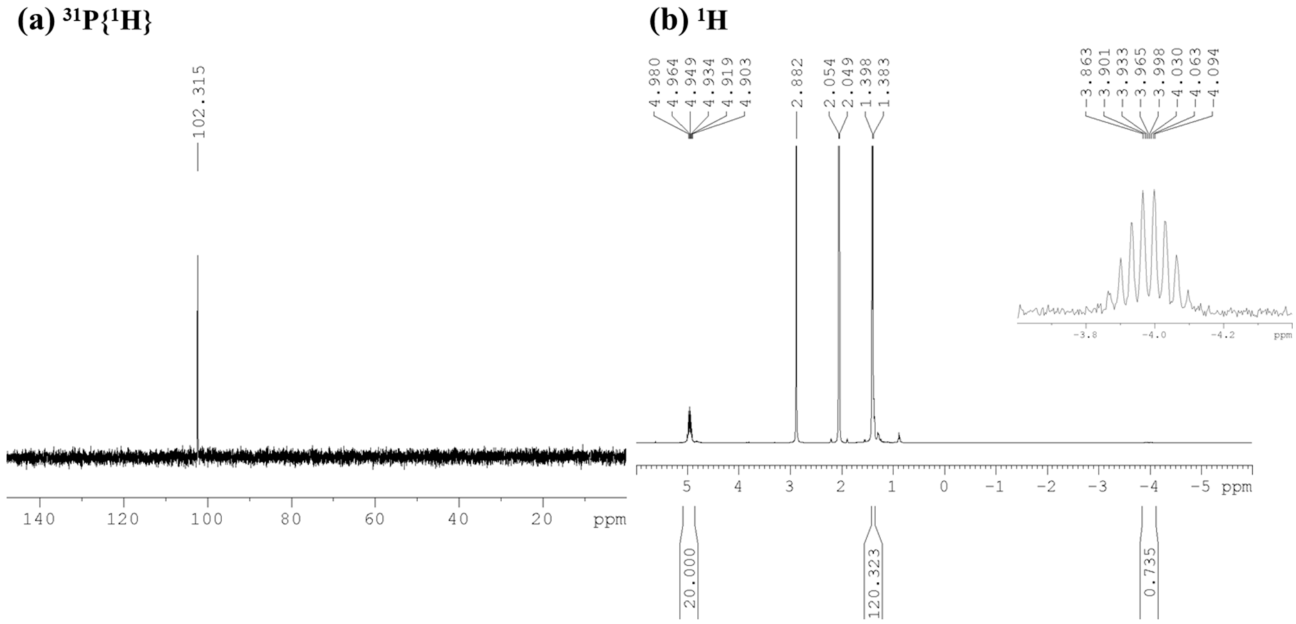

Figure S3. NMR spectrum of **PdHAg$_{13}$S** in $(CD_3)_2CO$. (a) $^{31}P\{^1H\}$ NMR (161.97 MHz) and (b) ^1H NMR (400 MHz) spectrum.

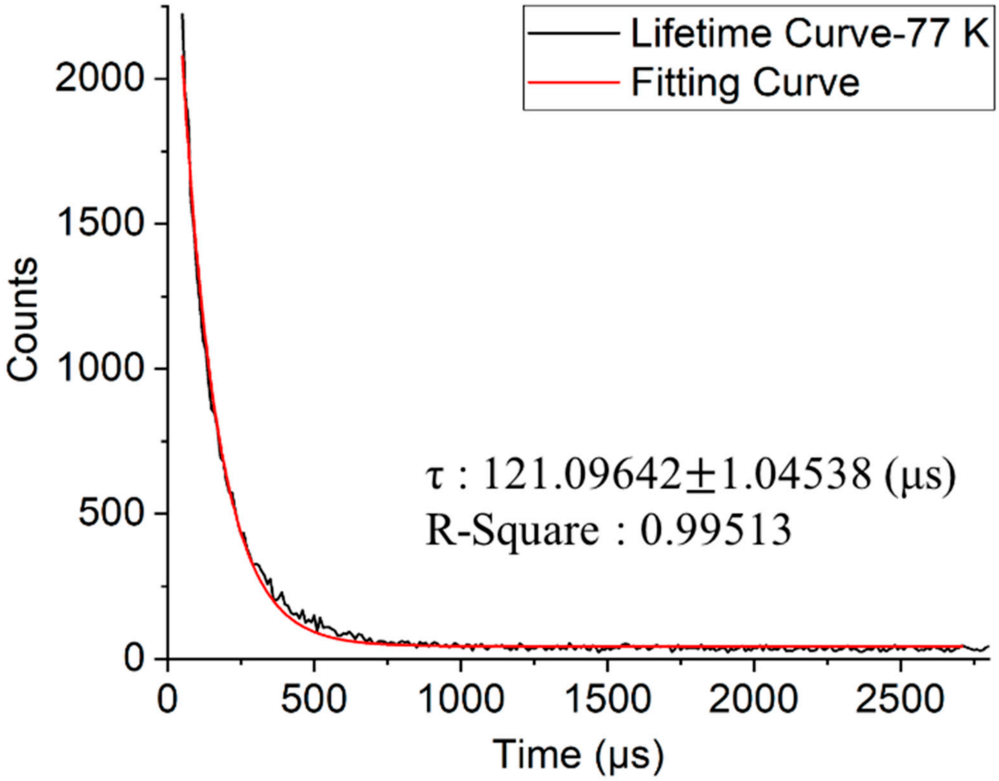

Figure S4. Time-resolved photoluminescence spectrum of **PdHAg$_{13}$S** in 2Me-THF at 77 K.

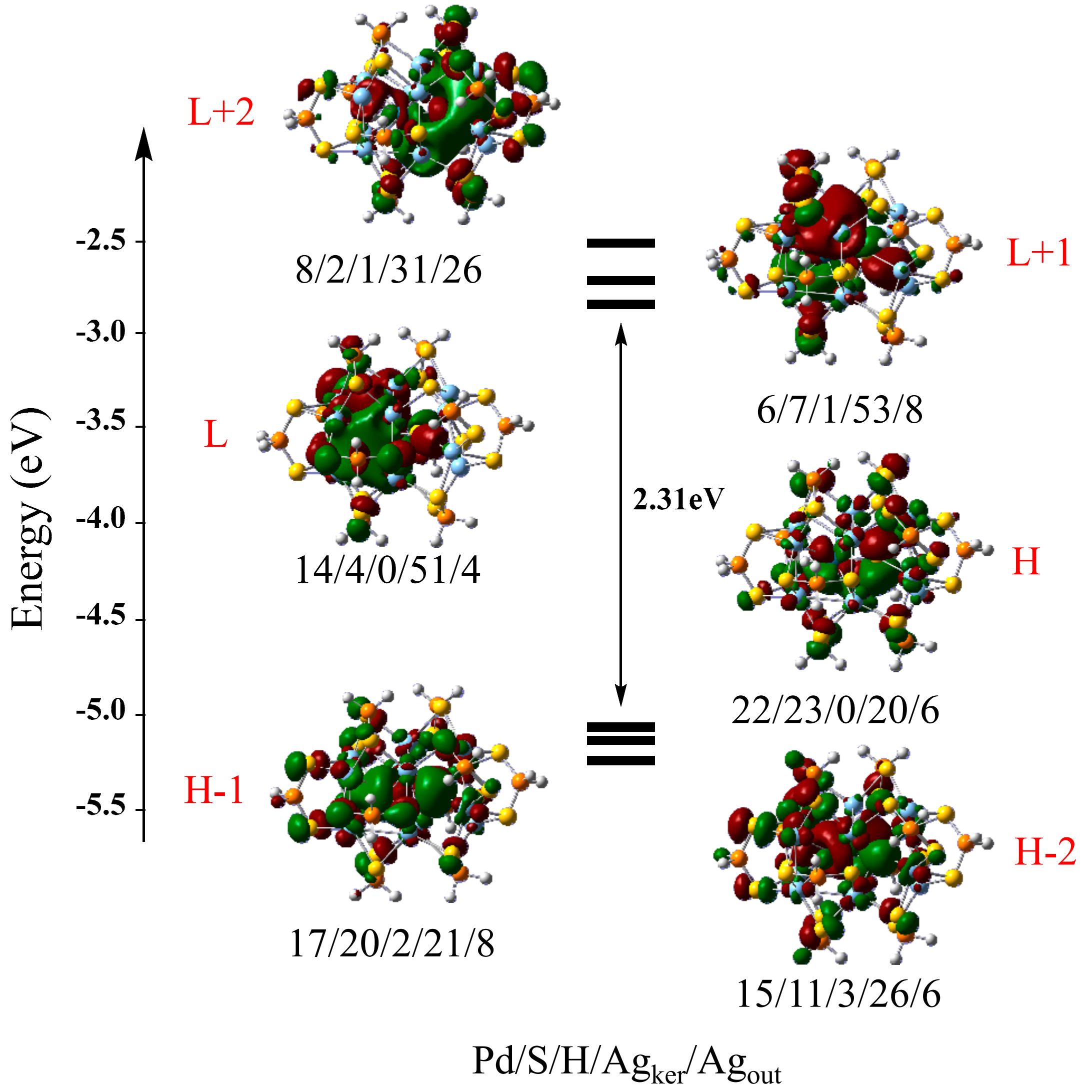

Figure S5. Kohn-Sham orbital diagrams of $HPdS@Ag_{13}(S_2PH_2)_{10}$. Numerical values indicate orbital localizations in % on the atoms indicated above (S = sulfide only).

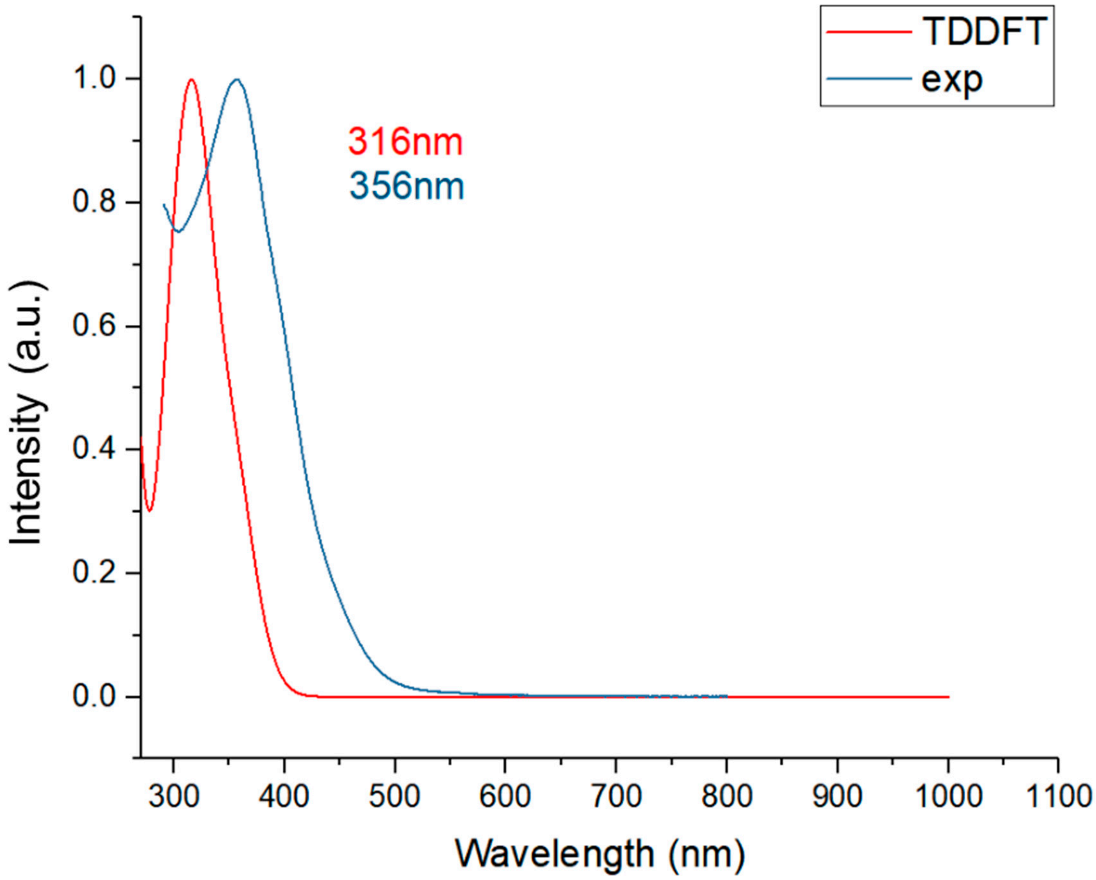

Figure S6. Experimental and TD-DFT-simulated UV-vis absorption spectrum.

Computational Details on optimized geometries for **PdHAg$_{13}$S**. (.xyz file)

**PdHAg$_{13}$S**	x	y	z
Ag	-2.982677	-0.118991	-2.038523
Ag	-2.998653	-2.075923	0.366051
Ag	-3.171839	0.287546	2.248361
Ag	-3.245849	2.144686	-0.090884
Ag	-0.068108	0.498622	2.776014
Ag	-0.099853	2.828913	0.045532
Ag	0.094092	1.043838	-2.354171
Ag	-0.336519	-1.950926	-2.026706
Ag	0.372646	-2.204954	1.31612
Ag	3.610593	-2.210032	1.134459
Ag	3.125864	1.166579	1.669574
Ag	3.08092	1.909941	-1.515082
Ag	2.995564	-1.268018	-1.775807
Pd	-0.99031	0.039095	0.080288
S	1.324088	0.059269	0.015217
H1M	-2.653044	-0.010666	0.106458
S	-5.35393	-1.116802	1.553516
S	-5.366462	1.021835	-1.351703
S	-3.820044	2.871997	2.410318
S	-0.260289	3.217411	2.672298
S	-1.938589	-0.527457	4.395956
S	-1.76534	-3.456919	2.350493
S	-3.423803	-4.214985	-1.017777
S	-2.443838	-2.085628	-3.675977
S	-2.068861	4.1451	-1.279369
S	-2.045158	1.679369	-3.854557
S	2.011726	4.193086	-0.913943
S	4.340488	3.46377	1.633347
S	2.229581	0.584488	4.140406

S	2.242981	-2.880614	3.205661
S	3.900919	-3.635092	-0.983427
S	0.355516	-4.139524	-0.681337
S	1.989603	1.901081	-4.061136
S	1.628913	-1.634757	-3.986037
S	5.395124	-0.390255	1.589306
S	5.119313	0.362963	-1.905852
P	-6.284132	-0.038609	0.11885
P	-2.154419	3.820016	3.066268
P	-1.906918	-2.545977	4.143027
P	-3.611576	-3.572626	-2.897701
P	-2.071649	3.602673	-3.235896
P	3.03398	4.712539	0.78006
P	2.674029	-1.352	4.482934
P	2.220194	-4.786484	-1.090174
P	1.859793	0.113084	-4.992402
P	6.155741	0.286711	-0.160258
H	2.059437	5.158046	1.713258
H	3.698324	5.931506	0.478523
H	6.66651	1.584718	0.093486
H	7.341173	-0.44426	-0.435087
H	2.990148	-0.037	-5.839515
H	0.811779	0.191847	-5.946868
H	-0.999508	4.284621	-3.868695
H	-3.194139	4.229859	-3.837157
H	-7.177996	0.857603	0.762906
H	-7.200258	-0.912839	-0.523342
H	-3.054707	-3.055067	4.805091
H	-0.867881	-3.048365	4.970228
H	2.091231	-1.716682	5.725335
H	4.055454	-1.438589	4.798203

H	2.449663	-5.934493	-0.287048
H	2.227399	-5.357313	-2.389883
H	-3.451157	-4.681167	-3.770904
H	-4.953804	-3.205507	-3.189003
H	-2.255817	5.18085	2.674799
H	-2.265712	3.931095	4.476855
